# Supplementary material for: A Green Solution for the Rehabilitation of Marginal Lands: The Case of Lablab purpureus (L.) Sweet Grown in Technosols
Source: Plants (Basel). 2023 Jul 18;12(14):2682. doi: 10.3390/plants12142682 (PMC10385650; doi:10.3390/plants12142682)
Supplement: Supplementary file 1 [file plants-12-02682-s001.zip › plants-2431144-supplementary.pdf]

Supplementary Materials:

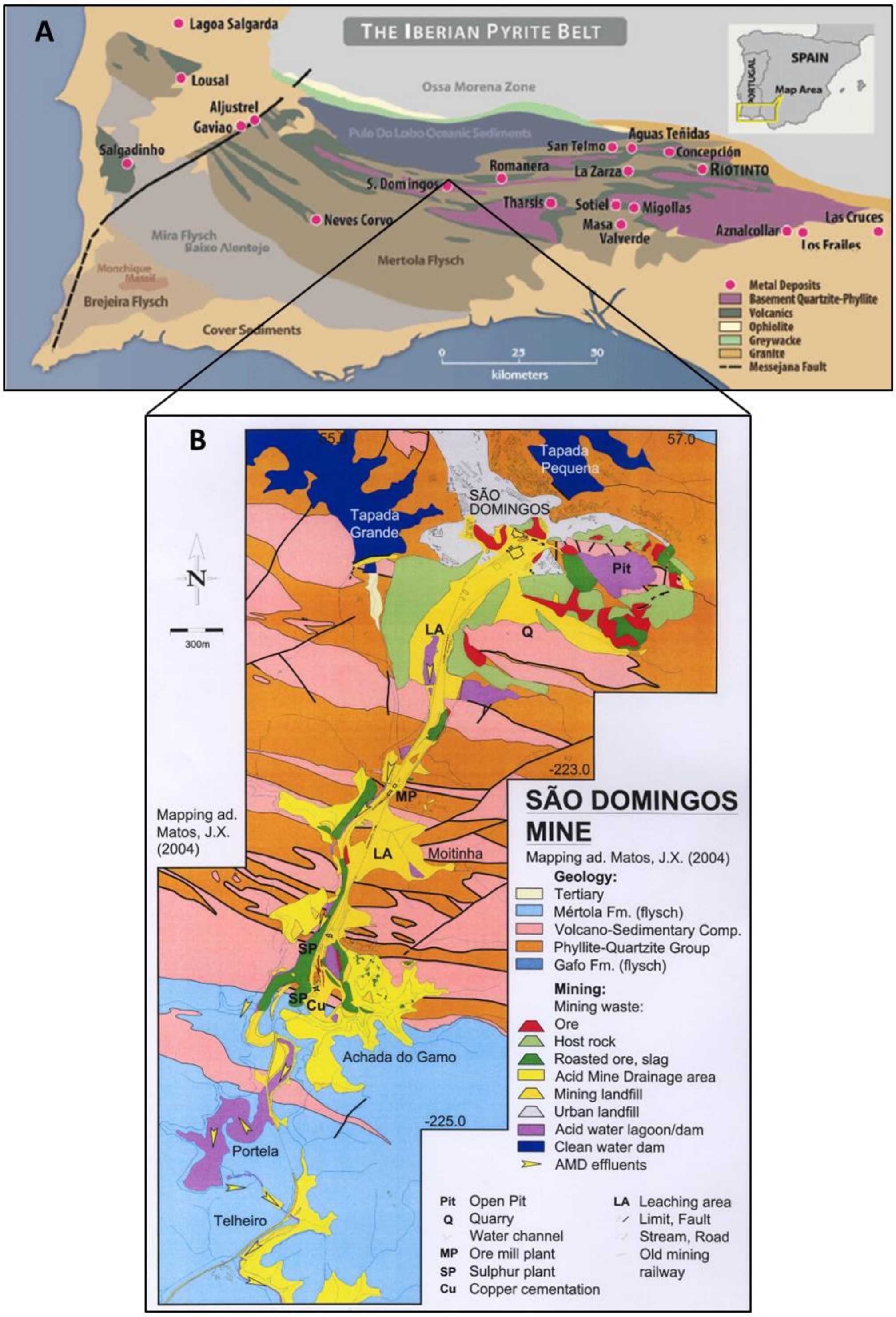

Figure S1. Location and geology of the Iberian Pyrite Belt in the Iberian Peninsula and of some of its most important mines (Ayterra SL®) (A), and geological and mining map of the São Domingos mine (extracted from [124]) (B).
